# Diagnosis of severe anaemia and positive antibody screening as consequences of pre-analytical error

**DOI:** 10.5937/jomb0-42844

**Published:** 2023-08-25

**Authors:** Marta Krygowska-Okrój, Katarzyna Kurpierz, Agnieszka Ćwiklińska

**Affiliations:** 1 Medical University of Gdańsk, Department of Clinical Chemistry, Gdańsk, Poland; 2 Medical University of Gdańsk, Department of Medical Laboratory Diagnostics, Gdańsk, Poland

**Keywords:** diagnostic error, hemoglobin, indirect antiglobulin test, phlebotomy, pre-analytical phase, dijagnostička greška, hemoglobin, indirektni antiglobulinski tst, flebotomija, pre-analiitička faza

## Abstract

The pre-analytical phase is the principal source of errors in laboratory medicine and continues to pose a challenge to laboratory professionals. We present the case of a 73-yearold female patient with a very low hemoglobin level (69 g/L) and positive indirect antiglobulin test result that indicates the key role of phlebotomy as an important error-prone process in which mistakes can have serious consequences for the patient's diagnosis and treatment. We conclude that there is still an urgent and continuous need to provide educational activities for healthcare professionals involved in blood collection, improve blood collection guideline adherence, and eliminate the errors which can affect diagnosis and treatment, thus jeopardising patient safety.

## Abstract (sr)

Pre-analitička faza je glavni izvor grešaka u laboratorijskoj medicini i nastavlja da predstavlja izazov za profesionalce u laboratoriji. Predstavljamo slučaj 73-godišnje pacijentkinje sa veoma niskim nivoom hemoglobina (69 g/L) i pozitivnim rezultatom indirektnog antiglobulinskog testa koji ukazuje na ključnu ulogu flebotomije kao važnog procesa sklonog greškama u kojem greške mogu imati ozbiljne posledice po dijagnozu i lečenje pacijenta. Zaključujemo da i dalje postoji hitna i stalna potreba da se obezbede edukativne aktivnosti za zdravstvene radnike koji se bave prikupljanjem krvi, da se poboljša poštovanje smernica za prikupljanje krvi i eliminišu greške koje mogu da utiču na dijagnozu i lečenje i na taj način ugroze bezbednost pacijenata.

## Introduction

A 73-year-old female patient was admitted to the emergency department and a correctly labeled EDTA blood sample obtained from the patient was delivered to the hospital laboratory at 1 pm. In accordance with the medical order, cell blood count (CBC) was performed and the hemoglobin (Hb) level was determined to be 69 g/L (reference range: 120 – 160 g/L), indicating severe aneamia. The physician ordering the examination was informed about the very low Hb result obtained.

Anaemia is one of the most prevalent disorders, particularly in the elderly population [1]. It is one of the main causes of hospital admission and prolongation of hospital stay, impairment of patient quality of life, and patient mortality. Thus, effective treatment of the condition is of great importance, and different methods of treatment appropriate for the patient’s condition are used, including iron therapy, erythropoiesis-stimulating agents, and blood transfusion [2].

Blood transfusion is a rapid and effective option for correcting anaemia and, in many patients (especially those with a very low Hb level), the only viable treatment option. However, transfusion can have important adverse effects, such as transmission of infectious diseases, immune-type reactions, and cardiopulmonary and thromboembolic complications. It also requires pre-transfusion laboratory testing that includes ABO and RH(D) blood group typing, an immune antibody screening with an indirect antiglobulin test (IAT) aiming to detect potentially clinically significant RBC antibodies, and *in vitro* compatibility testing (cross-matching) [3].

According to the current guidelines and restrictive transfusion strategy, the threshold for red blood cell (RBC) transfusion in hospitalized hemodynamically stable adult patients is Hb level of 70 g/L [4]. The patient met this criterion and pre-transfusion testing was ordered.

Subsequently, newly taken and correctly labelled blood samples were delivered to the transfusion-service unit of the laboratory at 2:30 pm for pre-transfusion testing. The test results are presented in Table 1. As immune antibodies were detected, the physician ordering the examination was informed about the test results and the need to collect new blood samples, so consultation tests could be performed in the Regional Blood Donation and Transfusion Centre (hereinafter Regional Centre).

**Table 1:** The results of the reactions for blood group testing and antibody screening. The blood grouping was performed with the microcolumn automated gel method using a Bio-Rad DiaMed Classic ID-Gelstation Analyzer with ID-Card DiaClon ABO/D (DVI+, DVI-) + Reverse Grouping (Blood grouping) and ID-Card LISS/Coombs (IAT).<br>*ID-DiaCell ABO; **ID-DiaCell I-II-III.

	Monoclonal reagents				Reference<br>blood cells*	
Blood<br>grouping	anti-A	anti-B	anti-DVI-	anti-DVI+	A1	B
	4	0	0	0	0	4
Indirect<br>antiglobulin<br>test (IAT)	Reference blood cells**					
	I	II	III			
	0	0	1			

The new blood samples were taken after the patient was transferred to the internal ward. EDTA and clotted sample to obtain the serum were delivered to the transfusion-service unit at around 8.30 pm, and after the samples and the documents were checked, they were immediately transported to the Unexpectedly, the test results did not confirm the presence of immune antibodies (Table 2).

**Table 2:** The blood group testing results. The consultative examination was performed using a fully automated system for ID-cards technology (IH-500, Bio-Rad) (ABO, RH, indirect antiglobulin test (IAT), enzymatic test) and manual tube testing (IAT, NaCl).

	Transfusion-service<br>unit of the hospital<br>laboratory	Regional Blood<br>Donation and<br>Transfusion Centre<br>(the consultative<br>examination)
Blood group	A RH(D)-negative	A RH(D)-negative
Immune<br>antibodies	Detected. Control<br>at Regional Blood<br>Donation and<br>Transfusion Centre<br>recommended.	Not detected.

In the following day at 6 am, the EDTA sample was delivered to the hospital laboratory for hematological analysis. The Hb result was 111 g/L. The next examination performed the same day showed similar Hb level: 107 g/L (Table 3). Thus, it was concluded that the result of 69 g/L was falsely decreased. The transfusion was cancelled.

**Table 3:** Cell blood count (CBC) results. Analysis was performed using 5-diff Auto Hematology Analyzer (Mindray BC-5380). Hb – hemoglobin, Ht – hematocrit, RBC – red blood cell, WBC – white blood cell, PLT – platelets.

	Sample 1<br>(Day 1,<br>delivered<br>to the<br>laboratory<br>at 1 pm)	Sample 2<br>(Day 2,<br>delivered<br>to the<br>laboratory<br>at 6 am)	Sample 3<br>(Day 2,<br>delivered<br>to the<br>laboratory<br>at 8 pm)	Reference<br>values
Hb (g/L)	69	111	106	120-160
Ht (%)	19.2	31.0	28.7	33-46
RBC<br>(x10^12^/L)	2.18	3.51	3.28	4.0-5.2
WBC<br>(x10^9^/L)	8.24	10.77	12.96	4.0-10.0
PLT<br>(x10^9^/L)	288	493	488	150-400

The falsely decreased Hb level and falsely positive IAT test result indicated that the laboratory error have occurred. The occurrence of administrative, pre-analytical (haemolysis and clotting), and analytical errors was excluded. Thus, it was concluded that the blood samples obtained in the emergency department were most likely diluted and contaminated due to blood collection in the proximity of the intravenous cat heter (or from the catheter), since the patient re ceived infusions (metoclopramide, paracetamol and balanced crystalloid solution (Optilyte®)).

The pre-analytical phase continues to be the major source of errors in laboratory medicine, and most pre-analytical errors result from inappropriate specimen collection and handling [5]. The consequences of these errors can be misidentification, *in vitro* haemolysis, clotting, insufficient volume, dilution, and contamination of the samples, resulting in inability to perform the analysis or erroneous results which can lead to inappropriate clinical decisions [6].

In our patient the falsely decreased Hb level due to a pre-analytical error resulted in severe anaemia diagnosis. Unfortunately, none of the laboratory parameters determined from the first blood sample was very low, and they did not individually indicate the possibility of pre-analytical error. Thus, an unnecessary pre-transfusion procedure was carried out. The second ambiguous result was the presence of immune antibodies detected in the screening test that were not confirmed in further analysis. In general, when the antibody screen is positive, further testing by an extended panel is required to determine whether there is any specificity against major RBC antigens [7]. Thus, according to the Polish legal regulations, the consequence of ascertaining the presence of immune antibodies in a screening test was the sending of the samples to the Regional Centre for the consultative testing whose purpose is to identify the immune antibodies and select the appropriate blood for the patient [7].

The falsely positive screening test result indicates that the interference occurred, most likely due to the presence of drugs. Drugs can cause positive antiglobulin tests by different mechanisms [8]. The first is the formation of RBC autoantibodies, which react with the patient’s RBC and, usually, with most other RBC *in vitro*, even if the drug is not present. The other mechanisms are based on the reaction between the drug antibodies with RBC *in vitro* only in the presence of the drug. Namely, a drug can bind to the RBC membrane, and the antibody against the drug can combine with the drug on the membrane leading to IgG-sensitized RBC. The next mechanism involves the chemical modification of RBC by the drug, which leads to nonspecific reactions with different proteins. The drug and its specific antibody can also form an immune complex, which can attach to RBC membranes and activate the complement [8]. Unfortunately, we are not able to establish which infusion component interfered with the antibody screening test or by which mechanism. Nor we can exclude that the patient received another drug shortly before blood was collected for the screening test that could have interfered in that test. However, the obtained results indicate that the interference required the presence of a drug, and the drug was not present in the blood samples sent to the Regional Centre.

Interferences in pre-transfusion blood testing can cause potentially fatal errors or delay in supplying blood for transfusion [7]. In our case, paradoxically, the delay probably prevented an unnecessary transfusion, since it is likely that, in the absence of immune antibodies in the screening test, the blood for transfusion would have been delivered earlier and the transfusion would have been performed. Nevertheless, the pre-analytical error during blood collection caused very serious consequences: unnecessary laboratory examinations and the patient was at risk of having an unnecessary treatment procedure carried out with important adverse effects.

Phlebotomy, as a significant source of pre-analytical errors, has been observed by other researchers. Allen et al. [9] and Hengelveld et al. [10] presented cases in which the dilution or contamination of blood with infusion components had significant effects on serum electrolyte levels and other laboratory parameters. Plebani et al. [11] noticed an inappropriate transfusion case due to a specimen being diluted. In a cross-sectional multicentre survey study, Simundic et al. [12] showed that phlebotomy practices were the most critical extra-analytical activity. It should also be emphasised that blood collection errors are very difficult to detect and often go unrecognized since in many cases the sample collection is distant from the direct control of laboratory professionals, especially where inpatients are concerned [6] [13].

Thus, there is still an urgent and continuous need to provide educational activities for healthcare professionals involved in blood collection, improve blood collection guideline adherence, and eliminate the errors which can affect diagnosis and treatment as significantly as demonstrated by the presented case, thus jeopardizing patient safety

## Dodatak

### List of abbreviations

CBC, cell blood count;<br>IAT, indirect antiglobulin test;<br>Hb, hemoglobin;<br>Ht, hematocrit;<br>RBC, red blood cell;<br>WBC, white blood cell;<br>PLT, platelets.

### Acknowledgements

This work was supported by the Medical University of Gdansk grant no. ST 01-50023/0004938.

### Conflict of interest statement

All the authors declare that they have no conflict of interest in this work.
